# A practical guide to the implementation of artificial intelligence in orthopaedic research—Part 3: How orthopaedic research benefits from the implementation of artificial intelligence

**DOI:** 10.1002/jeo2.70481

**Published:** 2025-10-31

**Authors:** James A. Pruneski, Ayoosh Pareek, Bálint Zsidai, Jacob F. Oeding, Jonathan D. Hughes, Felix C. Oettl, Philipp W. Winkler, Thomas Tischer, Elmar Herbst, Alberto Grassi, Michael T. Hirschmann, Christophe Ley, Yinan Yu, Kristian Samuelsson, ESSKA Artificial Intelligence Working Group

**Affiliations:** ^1^ Department of Orthopaedic Surgery Tripler Army Medical Center Honolulu Hawaii USA; ^2^ Sports Medicine and Shoulder Service, Hospital for Special Surgery New York New York USA; ^3^ Department of Orthopaedics, Institute of Clinical Sciences, Sahlgrenska Academy University of Gothenburg Gothenburg Sweden; ^4^ Sahlgrenska Sports Medicine Center Göteborg Sweden; ^5^ Department of Orthopedics Skåne University Hospital Malmö/Lund Sweden; ^6^ Mayo Clinic Alix School of Medicine, Mayo Clinic Rochester Minnesota USA; ^7^ Department of Orthopaedic Surgery UPMC Freddie Fu Sports Medicine Center Pittsburgh Pennsylvania USA; ^8^ Balgrist University Hospital Zurich Switzerland; ^9^ Department for Orthopaedics and Traumatology Kepler University Hospital GmbH, Johannes Kepler University Linz Linz Austria; ^10^ Department of Orthopaedic Surgery Universitymedicine Rostock Rostock Germany; ^11^ Department of Orthopaedic and Trauma Surgery Malteser Waldkrankenhaus Erlangen Erlangen Germany; ^12^ Department of Trauma, Hand and Reconstructive Surgery University Hospital Muenster Muenster Germany; ^13^ IIa Clinica Ortopedica e Traumatologica, IRCCS Istituto Ortopedico Rizzoli Bologna Italy; ^14^ Department of Orthopaedic Surgery and Traumatology Kantonsspital Baselland Bruderholz Switzerland; ^15^ University of Basel Basel Switzerland; ^16^ Department of Mathematics University of Luxembourg Esch‐sur‐Alzette Luxembourg; ^17^ Department of Computer Science and Engineering Chalmers University of Technology Gothenburg Sweden; ^18^ Department of Orthopaedics Sahlgrenska University Hospital Mölndal Sweden

**Keywords:** artificial intelligence, machine learning, orthopaedics, research methods, sports medicine

## Abstract

Artificial intelligence (AI) encompasses the development of systems that can perform human‐like tasks, such as treatment guidance, decision‐making, pattern recognition and understanding language. Within AI, machine learning and deep learning play pivotal roles in diagnosis and outcome prediction, while natural language processing aids in synthesising large datasets from the electronic medical record. In orthopaedics, AI has demonstrated success in various areas, including image evaluation, surgical planning, outcome prediction, cohort identification and administrative tasks. The purpose of this manuscript was to provide an overview of the benefits of AI implementation within the field of orthopaedics. An additional goal was to address the challenges associated with producing high quality AI‐based research in a rapidly developing field.

**Level of Evidence:**

Level IV.

**Abbreviations**
ACL: anterior cruciate ligament
AI: artificial intelligence
DL: deep learning
EMR: electronic medical record
ML: machine learning
NLP: natural language processing
UCL: ulnar collateral ligament

## INTRODUCTION

The broad field of artificial intelligence (AI), first introduced in 1955 by McCarthy et al., encompasses the process of developing systems that perform traditionally human tasks, including decision‐making, pattern recognition and understanding language [44]. Shortly thereafter, machine learning (ML) was introduced as a subset of AI by which algorithms and statistical models were developed that allowed computers to learn and make predictions or decisions from provided data without explicit programming [2, 57]. Within ML, deep learning (DL) encompasses the use of neural networks for tasks involving large volumes of data for complex tasks including image and speech recognition [48]. Natural language processing (NLP) is another subset of AI increasingly used in healthcare research, which can often incorporate elements of ML and DL to allow computers to understand and interpret human language [4, 56, 86]. These applications are described more in depth in a previous work [85]. Across all medical specialities, medical practitioners, insurance providers and the medical device industry are adapting AI techniques to aid in diagnosing and treating pathology, facilitating patient engagement and adherence and streamlining administrative tasks [2, 12, 21, 81].

**Figure 1:**
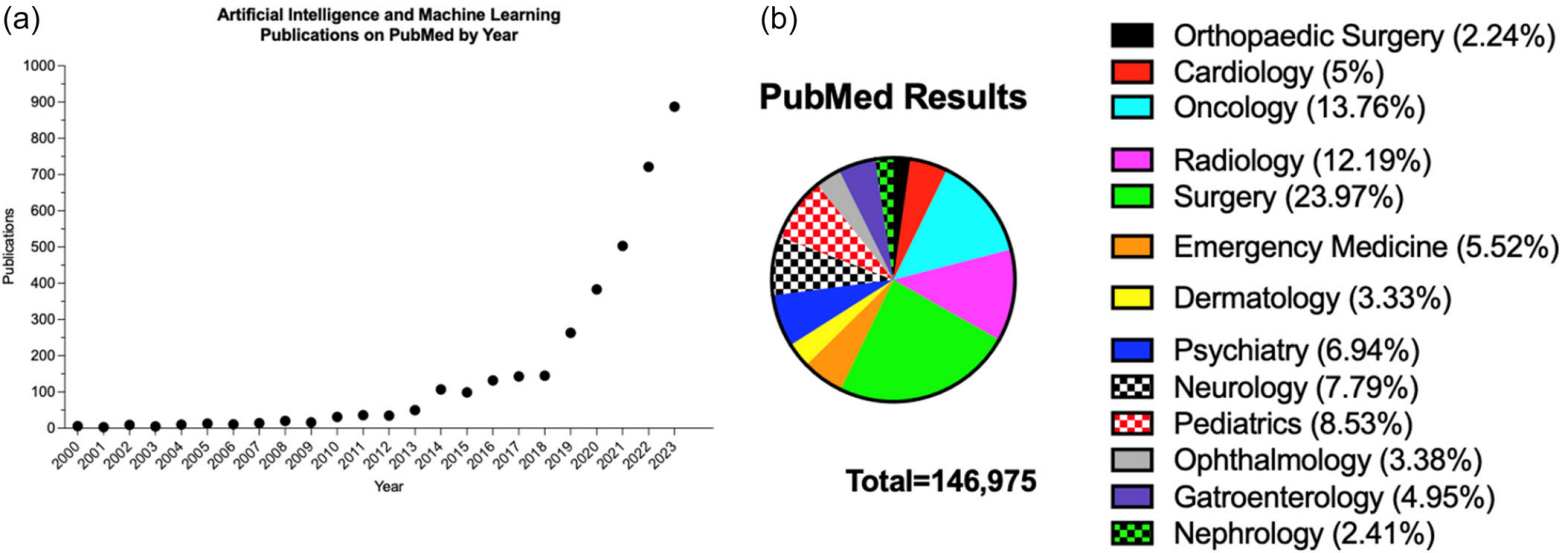
Artificial intelligence (AI) and machine learning publications on PubMed in (a) orthopaedic surgery, by year and (b) across a broad range of 13 medical specialities since 2000. Specific search queries used on 03 February 2024 were: (a) (‘AI’ OR ‘artificial intelligence’ OR ‘machine learning’) AND (‘orthopaedic’ OR ‘orthopaedic’), and (b) (‘AI’ OR ‘artificial intelligence’ OR ‘machine learning’) AND (Medical Specialty) AND (‘2000/01/01’(Date—Publication): ‘2024/02/03’(Date—Publication).

Concurrently, there is a growing body of literature regarding the use of AI in orthopaedic research. Orthopaedic researchers have demonstrated success in implementing AI for an array of tasks, including image evaluation, surgical planning and decision making, cohort identification, variable extraction and outcome prediction [10, 17, 35, 51, 63, 65, 68, 76, 82]. Of note, the orthopaedic field has been slower to implement AI techniques compared to other specialities such as oncology, general surgery and radiology, although the number of AI and ML publications within orthopaedics is increasing each year (Figure 1). These technological innovations provide enormous potential to improve surgical practice, research and education. On a larger scale, broader implementation of AI may further optimise systems‐level processes, including clinical documentation, scheduling, coding and billing and interactions with payers to facilitate greater efficiency in surgeon workflow. This work will explore the transformative potential of AI in orthopaedic research, focusing on its current applications and the prerequisites for high‐quality AI research in the field of orthopaedics.

## CURRENT STATE OF AI IN ORTHOPAEDIC RESEARCH

**Figure 2:**
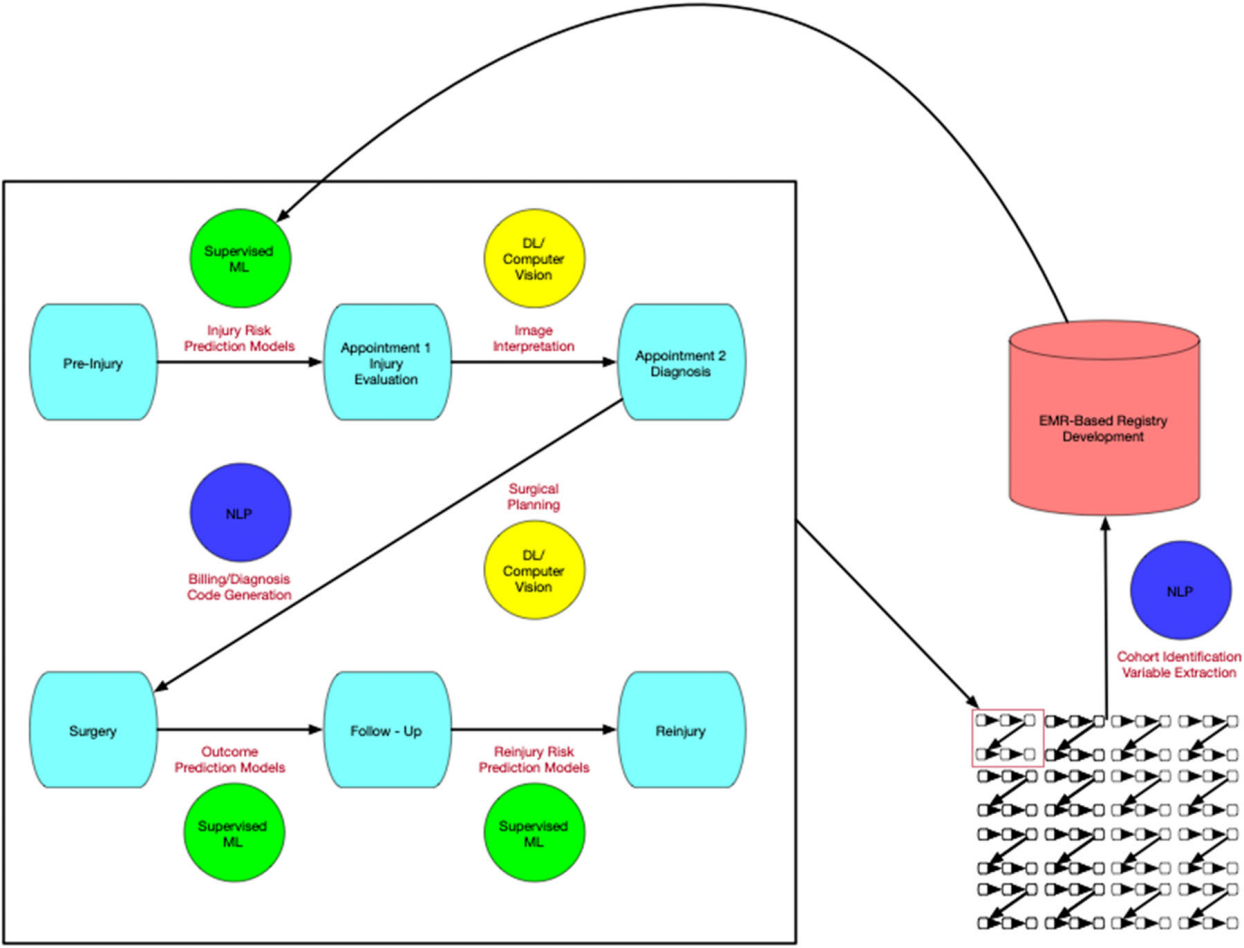
Areas for potential artificial intelligence (AI) applications throughout and after a patient‐care cycle. Beginning in a preinjury state, supervised machine learning (ML) models can be used to identify what puts patients at increased injury risk. Once injured, deep learning (DL) models can be used to identify pathology from provided images. Subsequently, DL models can assist with surgical planning and decision making (e.g., automated templating, automated measurements), and natural language processing (NLP) can be used to provide a list of applicable billing codes to the clinical encounter. After surgery, supervised ML models can be used to predict patient outcomes using patient and surgical variables, and similar models can predict re‐injury after surgery, capturing an entire patient‐care cycle. On a larger scale, NLP can be used to identify cohorts of interest from these care cycles and capture relevant variables to create real‐time registries efficiently and accurately. These registries can be used to train further models, allowing for a cycle of continuous improvement.

There has been a drastic increase in orthopaedic publications regarding the use of AI and ML in recent years [59]. The following review is not meant to be comprehensive, as such reviews already exist. This section serves to provide an overview of common themes of AI‐related research within the orthopaedic literature (Figure 2).

### Injury and outcome prediction

An important and clinically relevant use‐case for AI is in the prediction of injuries and outcomes. Within orthopaedics, researchers have found success in predicting several osseous and soft tissue pathologies (Table 1) [6, 20, 22, 47, 72, 75]. Additionally, authors have demonstrated success using AI to predict outcomes for an array of procedures, including ACL reconstruction, hip arthroscopy and knee and shoulder arthroplasty [13, 18, 32, 34, 41, 52, 78]. Arthroplasty dislocation calculators have also been developed with promising results [27, 46]. These analyses typically combine demographic, injury and surgical patient‐specific variables to predict patient‐reported outcomes, revision surgery or clinically meaningful improvement. Similarly, researchers combine various modifiable and nonmodifiable risk factors with the goal of determining what puts patients at higher risk for secondary or concomitant injuries that are clinically relevant but otherwise difficult to predict [47, 54].

**Table 1:** Examples of studies using artificial intelligence (AI) for injury risk prediction.

Author	Topic/aim	Conclusion/findings
Bulstra et al. [6]	Estimating probability of scaphoid fracture	The best classifier had a mean area under the receiver operating characteristic curve (AUC‐ROC) of 0.77. Additionally, the authors developed a decision rule with a sensitivity of 1.0, decreasing number of patients undergoing advanced imaging by 36% without missing a fracture.
Jauhiainen et al. [20]	Predicting risk of anterior cruciate ligament (ACL) injury based on physical screening tests	The best classifier had a mean AUC‐ROC of 0.63. Accordingly, some variables may assist with understanding causation, however, are insufficient to predict injury in practice.
Jurgensmeier et al. [22]	Predicting risk of secondary meniscus injury after ACL reconstruction	All 4 ML models outperformed traditional logistic regression. The best classifier had a mean AUC‐ROC of 0.79. Risk factors were identified for secondary meniscal injury.
Oeding et al. [47]	Predicting the presence of subscapularis tears based on preoperative exam and imaging	Using preoperative imaging factors, the model had an accuracy of 0.85, and identified five key imaging features associated with tear presence.
Whiteside et al. [75]	Identifying significant predictors of ulnar collateral ligament (UCL) reconstruction in MLB pitchers	The top performing model predicted UCL reconstruction with an accuracy of 0.75, Additionally, 6 key performance factors were identified as potential risk factors for UCL reconstruction.

### Imaging interpretation

One of the more apparent use‐cases for AI, particularly DL, is in the analysis and interpretation of imaging data. Importantly, the purpose of this implementation is not to replace the role of the physician in diagnosing musculoskeletal pathology, but rather to supplement the knowledge of the physician, identify patterns, minimise error and assist in the education of lower‐level trainees. Potential benefits of successful implementation include increasing efficiency in the diagnostic timeline, the ability to provide expert‐level interpretations in areas with limited access to care, and the potential to teach residents, fellows and students [15]. DL has been successfully implemented to aid in the diagnosis of several different pathologies across varying supspecialties (Table 2) [3, 35, 64, 66, 77, 82]. Additionally, implant identification tools have been developed which will aid future efforts for registry‐building and outcomes research, as well as potentially assist with timely planning for hardware removal and revision procedures [23, 31].

**Table 2:** Examples of studies using artificial intelligence (AI) for imaging interpretation.

Author	Topic/aim	Conclusion/findings
Bien et al. [3]	Development of a deep learning model to identify abnormalities and specific diagnoses from knee magnetic resonance imaging (MRI) studies	The model achieved an area under the receiver operating characteristic curve (AUC‐ROC) of 0.937, 0.965 and 0.847 for detecting abnormalities, anterior cruciate ligament (ACL) tears and meniscal tears, respectively. Providing model predictions significantly increased clinical experts' specificity in identifying ACL tears.
Karnuta et al. [23]	Development and testing of a deep learning system to classify total hip arthroplasty implants	The system discriminated 8 implant models with a mean AUC‐ROC of 0.991 in the external testing set. The software classified implants at a mean speed of 0.02 s per image.
Kunze et al. [31]	Development and testing of a deep learning system to classify total shoulder arthroplasty implants	The system discriminated 22 implant models with AUC‐ROCs between 0.994 and 1.000 in the independent testing set. The software classified implants at a mean speed of 0.079 s per image.
Shim et al. [64]	Development of a deep learning method to diagnose, classify and visualise rotator cuff tears	The neural network outperformed shoulder specialists with regards to binary accuracy (0.925 vs. 0.764) and specificity (0.86 vs. 0.61). Class activation maps were generated to provide information regarding the location and three‐dimensional size of the tear.
Suzuki et al. [66]	Development of a deep learning method to diagnose distal radius fractures	The model achieved an AUC‐ROC 0.993. The neural network based on anteroposterior and lateral radiographs had accuracy, sensitivity and specificity of 0.993, 0.987 and 1.00, respectively. The accuracy of the convolutional neural network was equal to or better than that of three orthopaedic hand surgeons.
Yamada et al. [77]	Development of a deep learning method to discriminate femoral neck fractures, trochanteric fractures and nonfracture	The average accuracy, sensitivity and specificity of the neural network were 0.98, 0.98 and 0.98, respectively. The accuracy of the model was comparable to, or statistically significantly better than, that of the orthopaedic surgeons.

### Surgical planning

One of the primary driving forces behind the growing interest in incorporating AI into the orthopaedic surgeon's workflow is the desire for increased efficiency and decreased time spent completing routine manual tasks. As such, incorporating AI into the surgical planning workflow is greatly desired, as much of this involves manual measurements and calculations, which can often vary between surgeons. Recent studies demonstrate successful implementation of AI platforms for automating an array of surgically relevant measurements (Table 3) [5, 11, 19, 37, 38, 40]. Surgical templating tools for arthroplasty procedures have also been developed with early success [62, 73]. The ability to have these measurements reliably calculated in a validated and reproducible manner can drastically improve the surgeon's workflow, while possible creating more predictable outcomes between surgeons.

**Table 3:** Examples of studies using artificial intelligence (AI) for surgical planning.

Author	Topic/aim	Conclusion/findings
Boileau et al. [5]	To determine whether 3D automated measurements of glenoid version and inclination are accurate and reliable	Concordance correlation coefficients between the automated approach and previously described measurement techniques ranged from 0.93 and 0.95 for glenoid version and was 0.78 for inclination.
Jang et al. [19]	Development of a deep learning platform to identify leg length discrepancy (LLD) landmarks and automate LLD measurements	Interclass correlation coefficients (ICC) varied from 0.73 and 0.98 for the six LLD methods. When comparing the methods for agreement, no combination had ICC > 0.90, and 53% of combinations had a poor ICC (<0.50).
Larson et al. [37]	Development of a deep learning platform to assess bone age	The mean difference between the neural network and radiologist bone age estimates was 0 years.
Larson et al. [38]	Development of a deep learning platform to identify LLD landmarks and automate LLD measurements	Anatomic landmarks were identified with sensitivity and specificity of 0.98 and 0.96, respectively. Correlation coefficients between radiologist and AI measurements were >0.99 for LLD measurements, and 0.98 and 0.86 for mechanical axis angle and pelvic tilt, respectively.
Rouzrokh et al. [62]	Development of a deep learning platform to generate synthetic postoperative hip arthroplasty radiographs.	The surgical validity of synthetic postoperative radiographs was higher than their real counterparts (by 0.8–1.1 points on 10‐point Likert scale), representing a potentially useful tool for arthroplasty templating.

### Administrative tasks

Outside of direct orthopaedic practice and research, there has also been early success in using AI to augment administrative tasks. Researchers have published the successful implementations of AI pipelines for the purposes of predicting operative time, length of hospital stay, costs and billing codes (Table 4) [24, 58, 68, 79]. Additionally, AI can assist in clinical documentation and consultation, allowing surgeons to see and treat more patients efficiently [16, 30]. Once validated, such models could optimise surgical scheduling, payment plans and administrative tasks in a way that minimises waste and costs and allowing the surgeon to spend more time on patient care.

**Table 4:** Examples of studies using artificial intelligence (AI) for administrative tasks.

Author	Topic/aim	Conclusion/findings
Karnuta et al. [24]	Development of a machine learning model to predict length of stay and cost after hip fracture	The model demonstrated 0.765 and 0.79 accuracy for length of stay and cost, respectively.
Ramkumar et al. [58]	Development of a deep learning model to predict length of stay, cost and discharge disposition after total knee arthroplasty	The model achieved an area under the receiver operating characteristic curve (AUC‐ROC) of 0.748, 0.828 and 0.761 for length of stay, costs and discharge disposition, respectively.
Tavabi et al. [68]	To assess the performance of common natural language processing techniques to predict current procedural terminology (CPT) codes from operative notes.	Traditional techniques, such as term frequency‐inverse document frequency (TF‐IDF), outperformed more computationally intensive transformer models, with a mean AUC‐ROC of 0.96 and accuracy of 0.97 when assessing the 100 most common musculoskeletal CPT codes.
Yeo et al. [79]	To assess the performance of different machine learning models in predicting operative time for patients undergoing total knee arthroplasty	The best performing model (neural network) achieved an AUC‐ROC of 0.82. Additionally, several factors were found to be predictive of surgical operative time.

## THE BENEFITS OF AI IN ORTHOPAEDIC RESEARCH

Modern increases in computing power and the vast amount of patient data available through the electronic medical record (EMR) have paved the way for the rapid growth of AI and ML research within orthopaedics. While ML theory has existed for over five decades, the biomedical literature has predominantly utilised traditional statistical methods in analysing patient data. In general, statistical methods are ‘top‐down’ approaches, in that a model and distribution are assumed, and unknown model parameters are estimated from the data [39]. In contrast, ML methods are ‘bottom‐up’ approaches, in which a model is developed through a standardised process with prediction or classification as the primary goal [39, 57]. Within ML, a range of models exist that vary in both complexity and explainability and can be tailored to suit specific tasks [55]. In general, explainability is sacrificed for predictive power when moving from models such as decision trees toward deep neural networks. ML models have demonstrated improved predictive ability compared to traditional regression in an array of clinical scenarios, including detecting osteoarthritis and predicting professional sports injuries [29, 42, 75]. Importantly, however explainability is paramount in healthcare AI implementation where clinical decisions have direct patient impact, as the ‘black box’ nature of advanced ML models can undermine trust among healthcare professionals, potentially perpetuates biases and complicate error correction [8, 84]. While complex models like neural networks may outperform simpler models in prediction tasks, their reduced transparency poses significant challenges [8, 84]. Achieving the optimal balance between predictive power and interpretability remains essential for responsible AI adoption in medical settings.

A key benefit of AI to orthopaedic research is its significant potential to augment the conduction of inductive research, by aiding in the creation of new hypotheses. Unsupervised ML techniques, such as clustering and principal components analysis, have been used in orthopaedics to identify subtle patterns and structures from high‐dimensional datasets [14]. For example, groups have identified outcome patterns in patients undergoing spinal deformity surgery, orthopaedic trauma surgery and total joint arthroplasty [1, 9, 60]. Additionally, clustering and principal component analyses have been used in kinematic studies to identify patterns in patients and associate those patterns with risk for bone stress injury risk and limited mobility after arthroplasty [43, 80]. By identifying these patterns in a timely manner, physicians are afforded the opportunity to tailor personalised treatment plans to optimise outcomes in patients. This ability to identify patterns and then create hypotheses or enact actionable plans is unique to ML and poses an exciting frontier for research in the era of ‘big data’.

For applications of AI in orthopaedic surgery to continue to evolve, a critical step will be the creation of large registries of data that can be built upon and utilised for multiple applications [36]. While these registries can be used to create novel ML and DL algorithms with significant predictive ability, AI models can also assist in the registry creation process itself. For example, AI can automate the cohort identification process by using unstructured clinical documentation from the EMR [67, 68, 69, 70]. This process has been historically labour intensive, logistically and financially expensive and plagued by error. Once the cohort is identified, variables of interest can be extracted from the EMR for the purpose of registry‐based research [54, 67, 76]. Furthermore, classification algorithms that can label imaging data (e.g., implant type, radiographic measurements) have the potential to add to the pool of data used to train ML models. These combined imaging and tabular data registries have demonstrated far greater predictive ability compared to tabular data‐trained models alone [27]. Without question, the ability to reliably create a registry that can be used for multiple clinical investigations may greatly improve the quality of research in the field of orthopaedics and will be a necessary step for algorithms to evolve beyond basic tasks such as fracture or tear detection.

Generative AI is an area of growing interest, as the ability to synthesise realistic data would benefit model training, testing and validation while protecting patient privacy. This is critical to the future of research, as DL performance has been shown to improve with large, diverse, high‐quality training datasets [74]. Within orthopaedics, researchers have demonstrated success in creating high‐quality pelvis radiographs, as well as anonymising existing radiographs, and improving image quality with DL [26, 28, 83]. Generative AI can also minimise barriers to collaboration in orthopaedics by minimising language barriers within academic writing [25, 50].

As previously mentioned, meaningful implementation of AI research in orthopaedics should augment the physician's workflow in a reliable way. AI, as opposed to statistics, is required to meet the complex demands of modern practice. The ability of these models to handle large amounts of nonlinear data, while being able to be fine‐tuned to specific practice scenarios makes them ideal to be used in an array of clinical scenarios. As the emphasis on personalised, precision medicine continues to increase, surgeons and researchers will begin to lean increasingly on these more complex models to improve efficiency and outcomes.

## CHALLENGES AND REQUIREMENTS FOR HIGH QUALITY AI RESEARCH

While the drastic increase in AI‐based orthopaedic literature demonstrates increased access to, interest in, and understanding of technology, the current state of AI within our field is not without pitfalls and challenges. Primarily, these models require high‐quality data, interdisciplinary collaboration and validation, appropriate context and interpretability prior to wide‐scale implementation for clinical practice. In their work, Cabitza et al. highlighted four key consequences of ML in medicine: reduced physician skills, the demise of context, the intrinsic uncertainty of clinical medicine, and uninterpretable output [7]. The authors highlighted examples of studies in which physicians demonstrated decreased diagnostic accuracy and sensitivity when analysing results that were annotated with inaccurate computer‐generated results, suggesting an overreliance on technology [7, 53, 71]. To avoid this potentially serious consequence for patient care, developers of AI models should define a priori whether the purpose of the model is to (1) augment clinician performance by performing tasks that humans are either not able to perform or for which AI may be better suited to perform, such as recognising unique patterns in images or large amounts of data, or (2) increase clinician efficiency by assisting with relatively routine tasks. DL models should be trained specifically for the stated purpose (i.e., trained on fractures that are commonly missed by humans if the goal is to augment clinician performance or trained on routine fractures if the goal is to improve workflow efficiency), and this purpose should be clearly communicated to users. Additionally, the data utilised in these models should be scrutinised for quality, as high amounts of missing data and nonrepresentative samples may cause performance bias, leading to limited generalisability [33, 49, 57]. These observations strengthen the notion that AI should augment, not replace, the work of the physician/surgeon, and that the user should understand, generally, how the model works, when it may be applied, and what limitations exist.

Importantly, even with high‐quality data, the models should still undergo external validation on other population data, as well as prospective evaluation prior to clinical implementation [57, 59]. This is one of the key tenants for high‐quality AI research, proposed by Ramkumar et al. [59]. Their work also proposed that inappropriate vernacular, repackaging registry data, overstating the ‘black‐box phenomenon’, and withholding full model code were key concerns about AI research within the field of orthopaedics. The black‐box phenomenon pertains to decreased interpretability of model methods, and the model's tendency to detect unconventional or out‐of‐context patterns that might not have previously been correlated or reported [7, 45]. Finally, the ethical considerations of these models must be considered prior to release. Patient privacy must be assured, training populations must be generalisable and representative to avoid bias, and data must be protected and defended both during and after model implementation [45]. While this is not a comprehensive evaluation of the challenges of integrating AI and ML applications to the field of orthopaedics, the aforementioned pitfalls are important to be aware of when evaluating potential tools or models proposed in the literature. Despite these challenges, the use of these models does not appear to be slowing down—or should it. Given that high‐quality data are collected, and a model is properly developed and evaluated for a specific purpose, AI models have the potential to significantly improve the clinician's ability to care for patients.

## PRACTICAL CONSIDERATIONS FOR AI‐DRIVEN RESEARCH

**Figure 3:**
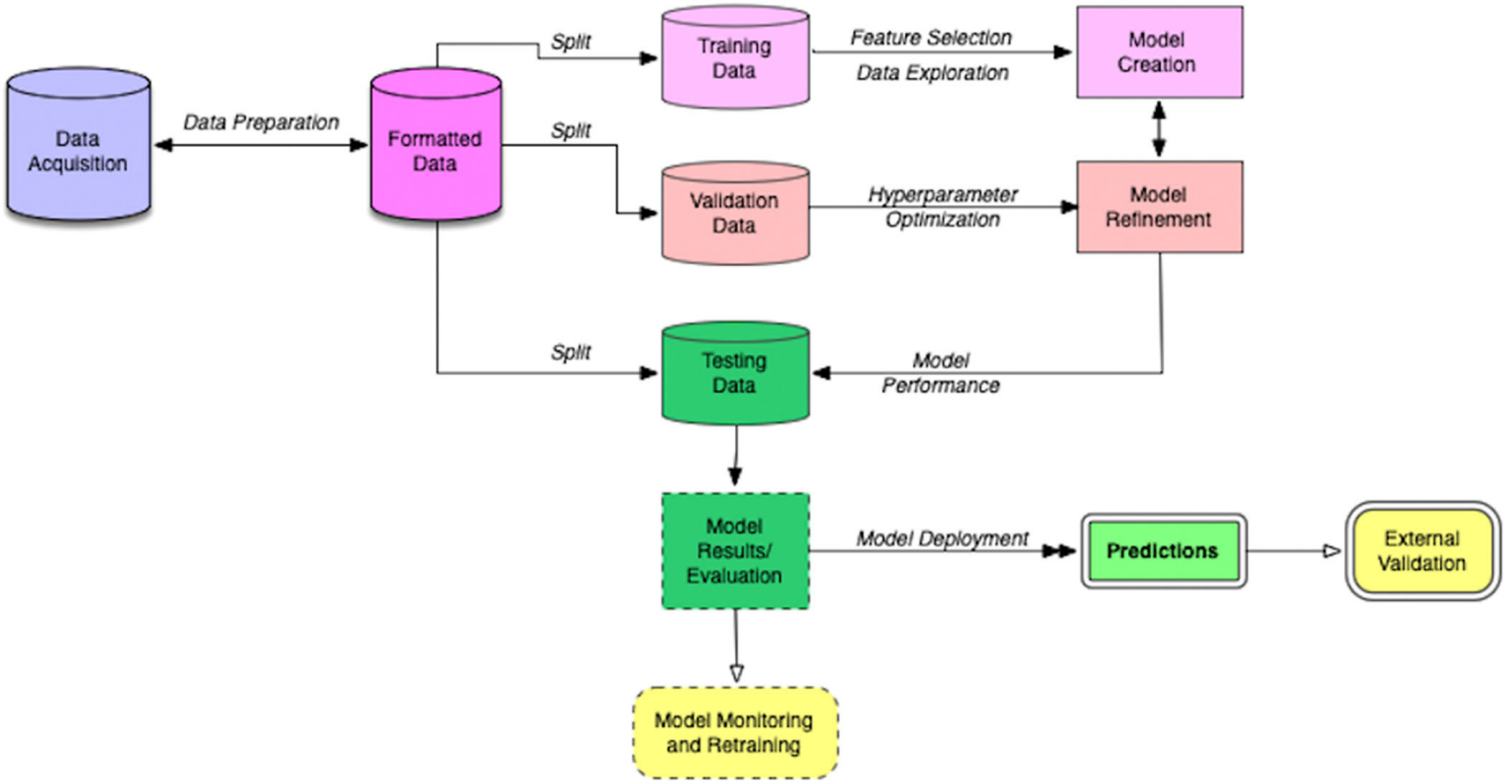
An illustrative process for crafting, assessing and implementing machine learning models. Following data preparation, the customary division into training, validation, and test sets is executed. The training dataset is predominantly employed for model creation and algorithm optimisation, while validation sets aid in fine‐tuning through hyperparameter selection. Subsequently, the model undergoes evaluation on blinded test datasets, ensuring unbiased assessment before transitioning into a functional predictive model. Ongoing monitoring and retraining are integral for maintenance, offering the flexibility of potential deployment to alternative sites for external validation. Figure used with permission from Pruneski et al. [57].

Equipped with a fundamental technical knowledge of AI, the orthopaedic researcher needs to consider additional factors to proceed with designing feasible AI‐driven research projects. Often, these projects are designed and executed by multidisciplinary teams, involving both clinical and technical specialists. When considering the next steps, orthopaedic researchers should make deliberate, well‐informed decisions about the type of domain‐specific research topics that can be investigated using AI. Data acquisition, management and processing for AI‐intensive research must be strategically planned. A plan must be established to assess the performance of AI systems, navigate the interpretability of AI‐driven research output and to validate the end‐product. A general approach to the implementation of AI for orthopaedic research is outlined in Figure 3. Subsequent parts of this learning series will focus on exploring these topics in more detail.

## FUTURE DIRECTIONS

The future of AI in orthopaedics is clearly promising given the rapid advances in technological and computational power. One can envision a scenario in which at any stage of a clinical encounter, from scheduling to diagnosis to surgical management, technology can autonomously augment the clinical workflow. Orthopaedic surgery could undergo a transformative shift as AI systems assist surgeons in planning procedures with unparalleled precision, optimising implant selection and reducing surgical risks. Generative AI may aid in the curation of large radiographic datasets, which in turn can allow for high‐quality diagnostic models. Additionally, generative AI‐driven decision support tools are expected to aid surgeons in tailoring treatment plans to individual patient needs, improving outcomes and reducing recovery times. These advancements are set to redefine orthopaedic surgery and research, ushering in an era of personalised and efficient care for patients with musculoskeletal disorders and injuries. However, this requires interdisciplinary communication and teamwork, quality assurance and external validation and collaboration to ensure that models are accurate and applicable to diverse clinical scenarios. While many institutional and administrative barriers exist in completing multicentre studies and collaborating via data and code sharing, the field of orthopaedics stands to benefit substantially from such collaboration. Federated learning is one paradigm that seeks to address the problem of data governance and privacy, wherein algorithms are trained collaboratively without exchanging the data itself [61]. Similar approaches and paradigms that minimise barriers to collaboration will allow AI applications to more broadly and rapidly transform the medical landscape.

## CONCLUSION

The valuable role of AI in orthopaedics is underscored by its ability to handle large and complex data and provide predictive power that surpasses traditional statistical methods. Researchers have demonstrated success in an array of clinical scenarios, from optimising surgical workflow to planning surgery and predicting outcomes. While there are challenges to be addressed, including data quality, validation and ethical considerations, AI's potential to improve clinical practice is undeniable. The future of AI in orthopaedics is promising, with the potential for autonomous clinical support, precision surgical planning and personalised patient care. To harness these benefits, interdisciplinary collaboration, quality assurance and external validation are essential. As such, the integration of AI into orthopaedics is advocated for its potential to advance patient care and our understanding of musculoskeletal pathology.

## AUTHOR CONTRIBUTIONS

All listed authors have contributed substantially to this work. James A. Pruneski, Ayoosh Pareek, Bálint Zsidai, Jacob F. Oeding, Philipp W. Winkler and Jonathan D. Hughes performed literature review, and primary manuscript preparation. Editing and final manuscript preparation was performed by Thomas Tischer, Felix C. Oettl, Elmar Herbst, Alberto Grassi, Michael T. Hirschmann, Christophe Ley, Yinan Yu and Kristian Samuelsson. All authors read and approved the final manuscript.

## CONFLICT OF INTEREST STATEMENT

Kristian Samuelsson is a member of the Board of Directors in Getinge AB. Jacob F. Oeding is a consultant for Kaliber.ai. Jonathan D. Hughes and Thomas Tischer are associate editors for KSSTA. Jonathan D. Hughes receives educational funding from Stryker, Smith and Nephew, Arthrex, New Clip. Elmar Herbst is the Deputy Editor‐in‐Chief of KSSTA. Michael T. Hirschmann is the editor‐in‐chief of KSSTA, and a consultant for Medacta, Symbios and Depuy Synthes.

## ETHICS STATEMENT

The authors have nothing to report.

## Data Availability

The authors have nothing to report.

## References

[1] Ames CP , Smith JS , Pellisé F , Kelly M , Alanay A , Acaroğlu E , et al. Artificial intelligence based hierarchical clustering of patient types and intervention categories in adult spinal deformity surgery: towards a new classification scheme that predicts quality and value. Spine. 2019;44:915–926. 31205167 10.1097/BRS.0000000000002974

[2] Beam AL , Kohane IS . Big data and machine learning in health care. JAMA. 2018;319:1317–1318. 29532063 10.1001/jama.2017.18391

[3] Bien N , Rajpurkar P , Ball RL , Irvin J , Park A , Jones E , et al. Deep‐learning‐assisted diagnosis for knee magnetic resonance imaging: development and retrospective validation of MRNet. PLoS Med. 2018;15:e1002699. 30481176 10.1371/journal.pmed.1002699 PMC6258509

[4] Bobba PS , Sailer A , Pruneski JA , Beck S , Mozayan A , Mozayan S , et al. Natural language processing in radiology: clinical applications and future directions. Clin Imaging. 2023;97:55–61. 36889116 10.1016/j.clinimag.2023.02.014

[5] Boileau P , Cheval D , Gauci M‐O , Holzer N , Chaoui J , Walch G . Automated three‐dimensional measurement of glenoid version and inclination in arthritic shoulders. J Bone Jt Surg. 2018;100:57–65. 10.2106/JBJS.16.01122 29298261

[6] Bulstra AEJ , Buijze GA , Bulstra AEJ , Cohen A , Colaris JW , Court‐Brown CM , et al. A machine learning algorithm to estimate the probability of a true scaphoid fracture after wrist trauma. J Hand Surg. 2022;47:709–718. 10.1016/j.jhsa.2022.02.023 35667955

[7] Cabitza F , Rasoini R , Gensini GF . Unintended consequences of machine learning in medicine. JAMA. 2017;318:517–518. 28727867 10.1001/jama.2017.7797

[8] Caruana R , Lou Y , Gehrke J , Koch P , Sturm M , Elhadad N . Intelligible models for healthcare: predicting pneumonia risk and hospital 30‐day readmission. In: Cao L , editor. Proceedings of the 21th ACM SIGKDD international conference on knowledge discovery and data mining. Sydney, NSW, Australia: Association for Computing Machinery; 2015.

[9] Castillo RC , Huang Y , Scharfstein D , Frey K , Bosse MJ , Pollak AN , et al. Association between 6‐week postdischarge risk classification and 12‐month outcomes after orthopedic trauma. JAMA Surg. 2019;154:e184824. 30566192 10.1001/jamasurg.2018.4824 PMC6439663

[10] Chen X , Liu X , Wang Y , Ma R , Zhu S , Li S , et al. Development and validation of an artificial intelligence preoperative planning system for total hip arthroplasty. Front Med. 2022;9:841202. 10.3389/fmed.2022.841202 PMC8981237 35391886

[11] Cho B , Geng E , Arvind V , Valliani AA , Tang JE , Schwartz J , et al. Understanding artificial intelligence and predictive analytics: a clinically focused review of machine learning techniques. JBJS Rev. 2022;10(3):e21. 10.2106/JBJS.RVW.21.00142 35302963

[12] Davenport T , Kalakota R . The potential for artificial intelligence in healthcare. Fut Healthcare J. 2019;6:94–98. 10.7861/futurehosp.6-2-94 PMC6616181 31363513

[13] Domb BG , Ouyang VW , Go CC , Gornbein JA , Shapira J , Meghpara MB , et al. Personalized medicine using predictive analytics: a machine learning‐based prognostic model for patients undergoing hip arthroscopy. Am J Sports Med. 2022;50:1900–1908. 35536218 10.1177/03635465221091847

[14] Eckhardt CM , Madjarova SJ , Williams RJ , Ollivier M , Karlsson J , Pareek A , et al. Unsupervised machine learning methods and emerging applications in healthcare. Knee Surg Sports Traumatol Arthrosc. 2023;31:376–381. 36378293 10.1007/s00167-022-07233-7

[15] Fritz B , Fritz J . Artificial intelligence for MRI diagnosis of joints: a scoping review of the current state‐of‐the‐art of deep learning‐based approaches. Skeletal Radiol. 2022;51:315–329. 34467424 10.1007/s00256-021-03830-8 PMC8692303

[16] Gill K , Cacciamani G , Nabhani J , Corb J , Buchanan T , Park D , et al. A novel artificial intelligence platform to automate clinical consultation notes and enhance diagnostic efficiency in the outpatient clinic: a multi‐center, multi‐disciplinary, prospective randomized controlled trial. *medRxiv*; 2023. 10.1101/2023.06.26.232918792023.2006.2026.23291879

[17] Hall T , Wong JRY , Dirckx M , Rajesparan K , Rashid A . Pre‐operative arthritic glenoid assessment: 3D automated planning software versus manual multiplanar measurements of version and inclination. J Orthop. 2023;36:24–28. 36582547 10.1016/j.jor.2022.12.005 PMC9793237

[18] Hinterwimmer F , Lazic I , Langer S , Suren C , Charitou F , Hirschmann MT , et al. Prediction of complications and surgery duration in primary TKA with high accuracy using machine learning with arthroplasty‐specific data. Knee Surg Sports Traumatol Arthrosc. 2023;31:1323–1333. 35394135 10.1007/s00167-022-06957-w PMC10050062

[19] Jang SJ , Kunze KN , Bornes TD , Anderson CG , Mayman DJ , Jerabek SA , et al. Leg‐length discrepancy variability on standard anteroposterior pelvis radiographs: an analysis using deep learning measurements. J Arthroplasty. 2023;38:2017–2023.e3. 36898486 10.1016/j.arth.2023.03.006

[20] Jauhiainen S , Kauppi J‐P , Krosshaug T , Bahr R , Bartsch J , Äyrämö S . Predicting ACL injury using machine learning on data from an extensive screening test battery of 880 female elite athletes. Am J Sports Med. 2022;50:2917–2924. 35984748 10.1177/03635465221112095 PMC9442771

[21] Jiang F , Jiang Y , Zhi H , Dong Y , Li H , Ma S , et al. Artificial intelligence in healthcare: past, present and future. Stroke Vasc Neurol. 2017;2:230–243. 29507784 10.1136/svn-2017-000101 PMC5829945

[22] Jurgensmeier K , Till SE , Lu Y , Arguello AM , Stuart MJ , Saris DBF , et al. Risk factors for secondary meniscus tears can be accurately predicted through machine learning, creating a resource for patient education and intervention. Knee Surg Sports Traumatol Arthrosc. 2022;31:518–529. 35974194 10.1007/s00167-022-07117-w PMC10138786

[23] Karnuta JM , Murphy MP , Luu BC , Ryan MJ , Haeberle HS , Brown NM , et al. Artificial intelligence for automated implant identification in total hip arthroplasty: a multicenter external validation study exceeding two million plain radiographs. J Arthroplasty. 2023;38:1998–2003.e1. 35271974 10.1016/j.arth.2022.03.002

[24] Karnuta JM , Navarro SM , Haeberle HS , Billow DG , Krebs VE , Ramkumar PN . Bundled care for hip fractures: a machine‐learning approach to an untenable patient‐specific payment model. J Orthop Trauma. 2019;33:324–330. 30730360 10.1097/BOT.0000000000001454

[25] Kayaalp ME , Ollivier M , Winkler PW , Dahmen J , Musahl V , Hirschmann MT , et al. Embrace responsible ChatGPT usage to overcome language barriers in academic writing. Knee Surg Sports Traumatol Arthrosc. 2024;32:5–9. 38226673 10.1002/ksa.12014

[26] Khosravi B , Mickley JP , Rouzrokh P , Taunton MJ , Larson AN , Erickson BJ , et al. Anonymizing radiographs using an object detection deep learning algorithm. Radiol Artif Intel. 2023;5:e230085. 10.1148/ryai.230085 PMC10698585 38074777

[27] Khosravi B , Rouzrokh P , Maradit Kremers H , Larson DR , Johnson QJ , Faghani S , et al. Patient‐specific hip arthroplasty dislocation risk calculator: an explainable multimodal machine learning–based approach. Radiol Artif Intel. 2022;4:e220067. 10.1148/ryai.220067 PMC9745445 36523643

[28] Khosravi B , Rouzrokh P , Mickley JP , Faghani S , Larson AN , Garner HW , et al. Creating high fidelity synthetic pelvis radiographs using generative adversarial networks: unlocking the potential of deep learning models without patient privacy concerns. J Arthroplasty. 2023;38:2037–2043.e1. 36535448 10.1016/j.arth.2022.12.013 PMC12536427

[29] Kotti M , Duffell LD , Faisal AA , McGregor AH . Detecting knee osteoarthritis and its discriminating parameters using random forests. Med Eng Phys. 2017;43:19–29. 28242181 10.1016/j.medengphy.2017.02.004 PMC5390773

[30] Kumah‐Crystal Y , Pirtle C , Whyte H , Goode E , Anders S , Lehmann C . Electronic health record interactions through voice: a review. Appl Clin Inform. 2018;09:541–552. 10.1055/s-0038-1666844 PMC6051768 30040113

[31] Kunze KN , Jang SJ , Li TY , Pareek A , Finocchiaro A , Fu MC , et al. Artificial intelligence for automated identification of total shoulder arthroplasty implants. J Shoulder Elbow Surg. 2023;32:2115–2122. 37172888 10.1016/j.jse.2023.03.028

[32] Kunze KN , Kaidi A , Madjarova S , Polce EM , Ranawat AS , Nawabi DH , et al. External validation of a machine learning algorithm for predicting clinically meaningful functional improvement after arthroscopic hip preservation surgery. Am J Sports Med. 2022;50:3593–3599. 36135373 10.1177/03635465221124275

[33] Kunze KN , Orr M , Krebs V , Bhandari M , Piuzzi NS . Potential benefits, unintended consequences, and future roles of artificial intelligence in orthopaedic surgery research. Bone Jt Open. 2022;3:93–97. 35084227 10.1302/2633-1462.31.BJO-2021-0123.R1 PMC9047073

[34] Kunze KN , Polce EM , Ranawat AS , Randsborg PH , Williams RJ , Allen AA , et al. Application of machine learning algorithms to predict clinically meaningful improvement after arthroscopic anterior cruciate ligament reconstruction. Orthop J Sports Med. 2021;9:23259671211046575. 34671691 10.1177/23259671211046575 PMC8521431

[35] Kunze KN , Rossi DM , White GM , Karhade AV , Deng J , Williams BT , et al. Diagnostic performance of artificial intelligence for detection of anterior cruciate ligament and meniscus tears: a systematic review. Arthrosc J Arthrosc Rel Surg. 2021;37:771–781. 10.1016/j.arthro.2020.09.012 32956803

[36] Kunze KN , Williams RJ , Ranawat AS , Pearle AD , Kelly BT , Karlsson J , et al. Artificial intelligence (AI) and large data registries: Understanding the advantages and limitations of contemporary data sets for use in AI research. Knee Surg Sports Traumatol Arthrosc. 2024;32:13–18. 38226678 10.1002/ksa.12018

[37] Larson DB , Chen MC , Lungren MP , Halabi SS , Stence NV , Langlotz CP . Performance of a deep‐learning neural network model in assessing skeletal maturity on pediatric hand radiographs. Radiology. 2018;287:313–322. 29095675 10.1148/radiol.2017170236

[38] Larson N , Nguyen C , Do B , Kaul A , Larson A , Wang S , et al. Artificial intelligence system for automatic quantitative analysis and radiology reporting of leg length radiographs. J Digit Imaging. 2022;35:1494–1505. 35794502 10.1007/s10278-022-00671-2 PMC9261153

[39] Ley C , Martin RK , Pareek A , Groll A , Seil R , Tischer T . Machine learning and conventional statistics: making sense of the differences. Knee Surg Sports Traumatol Arthrosc. 2022;30:753–757. 35106604 10.1007/s00167-022-06896-6

[40] Lezak BA , Pruneski JA , Oeding JF , Kunze KN , Williams RJ , Alaia MJ , et al. Diagnostic performance of deep learning for leg length measurements on radiographs in leg length discrepancy: a systematic review. J Exp Orthop. 2024;11:e70080. 39530113 10.1002/jeo2.70080 PMC11551063

[41] Lu Y , Reinholz AK , Till SE , Kalina SV , Saris DBF , Camp CL , et al. Predicting the risk of posttraumatic osteoarthritis after primary anterior cruciate ligament reconstruction: a machine learning time‐to‐event analysis. Am J Sports Med. 2023;51:1673–1685. 37171158 10.1177/03635465231168139

[42] Luu BC , Wright AL , Haeberle HS , Karnuta JM , Schickendantz MS , Makhni EC , et al. Machine learning outperforms logistic regression analysis to predict next‐season NHL player injury: an analysis of 2322 players from 2007 to 2017. Orthop J Sports Med. 2020;8:2325967120953404. 33029545 10.1177/2325967120953404 PMC7522848

[43] Martin JA , Stiffler‐Joachim MR , Wille CM , Heiderscheit BC . A hierarchical clustering approach for examining potential risk factors for bone stress injury in runners. J Biomech. 2022;141:111136. 35816783 10.1016/j.jbiomech.2022.111136 PMC9773850

[44] McCarthy J , Minsky ML , Rochester N , Shannon CE . A proposal for the dartmouth summer research project on artificial intelligence, August 31, 1955. AI Magazine. 2006;27:12.

[45] Myers TG , Ramkumar PN , Ricciardi BF , Urish KL , Kipper J , Ketonis C . Artificial intelligence and orthopaedics: an introduction for clinicians. J Bone Jt Surg. 2020;102:830–840. 10.2106/JBJS.19.01128 PMC7508289 32379124

[46] Oeding JF , Lu Y , Pareek A , Marigi EM , Okoroha KR , Barlow JD , et al. Understanding risk for early dislocation resulting in reoperation within 90 days of reverse total shoulder arthroplasty: extreme rare event detection through cost sensitive machine learning. J Should Elbow Surg. 2023;32:e437–e450. 10.1016/j.jse.2023.03.001 36958524

[47] Oeding JF , Pareek A , Nieboer MJ , Rhodes NG , Tiegs‐Heiden CA , Camp CL , et al. A machine learning model demonstrates excellent performance in predicting subscapularis tears based on pre‐operative imaging parameters alone. Arthrosc J Arthrosc Rel Surg. 2024;40(4):1044–1055. 10.1016/j.arthro.2023.08.084 37716627

[48] Oeding JF , Williams RJ , Nwachukwu BU , Martin RK , Kelly BT , Karlsson J , et al. A practical guide to the development and deployment of deep learning models for the Orthopedic surgeon: part I. Knee Surg Sports Traumatol Arthrosc. 2023;31:382–389. 36427077 10.1007/s00167-022-07239-1

[49] Oettl FC , Pareek A , Winkler PW , Zsidai B , Pruneski JA , Senorski EH , et al. A practical guide to the implementation of AI in orthopaedic research, Part 6: how to evaluate the performance of AI research? J Exp Orthop. 2024;11:e12039. 38826500 10.1002/jeo2.12039 PMC11141501

[50] Ollivier M , Pareek A , Dahmen J , Kayaalp ME , Winkler PW , Hirschmann MT , et al. A deeper dive into ChatGPT: history, use and future perspectives for orthopaedic research. Knee Surg Sports Traumatol Arthrosc. 2023;31:1190–1192. 36894785 10.1007/s00167-023-07372-5

[51] Pasha S , Shah S , Newton P . Machine learning predicts the 3D outcomes of adolescent idiopathic scoliosis surgery using patient–surgeon specific parameters. Spine. 2021;46:579–587. 33821816 10.1097/BRS.0000000000003795

[52] Polce EM , Kunze KN , Fu MC , Garrigues GE , Forsythe B , Nicholson GP , et al. Development of supervised machine learning algorithms for prediction of satisfaction at 2 years following total shoulder arthroplasty. J Shoulder Elbow Surg. 2021;30:e290–e299. 33010437 10.1016/j.jse.2020.09.007

[53] Povyakalo AA , Alberdi E , Strigini L , Ayton P . How to discriminate between computer‐aided and computer‐hindered decisions: a case study in mammography. Med Decis Making. 2013;33:98–107. 23300205 10.1177/0272989X12465490

[54] Pruneski JA , Heyworth BE , Kocher MS , Tavabi N , Milewski MD , Kramer DE , et al. Prevalence and predictors of concomitant meniscal and ligamentous injuries associated with ACL surgery: an analysis of 20 years of ACL reconstruction at a tertiary care children's hospital. Am J Sports Med. 2024;52:77–86. 38164668 10.1177/03635465231205556

[55] Pruneski JA , Pareek A , Kunze KN , Martin RK , Karlsson J , Oeding JF , et al. Supervised machine learning and associated algorithms: applications in orthopedic surgery. Knee Surg Sports Traumatol Arthrosc. 2022;31:1196–1202. 36222893 10.1007/s00167-022-07181-2

[56] Pruneski JA , Pareek A , Nwachukwu BU , Martin RK , Kelly BT , Karlsson J , et al. Natural language processing: using artificial intelligence to understand human language in orthopedics. Knee Surg Sports Traumatol Arthrosc. 2022;31:1203–1211. 36477347 10.1007/s00167-022-07272-0

[57] Pruneski JA , Williams RJ , Nwachukwu BU , Ramkumar PN , Kiapour AM , Martin RK , et al. The development and deployment of machine learning models. Knee Surg Sports Traumatol Arthrosc. 2022;30:3917–3923. 36083354 10.1007/s00167-022-07155-4

[58] Ramkumar PN , Karnuta JM , Navarro SM , Haeberle HS , Scuderi GR , Mont MA , et al. Deep learning preoperatively predicts value metrics for primary total knee arthroplasty: development and validation of an artificial neural network model. J Arthroplasty. 2019;34:2220–2227.e1. 31285089 10.1016/j.arth.2019.05.034

[59] Ramkumar PN , Pang M , Polisetty T , Helm JM , Karnuta JM . Meaningless applications and misguided methodologies in artificial intelligence‐related orthopaedic research propagates hype over hope. Arthrosc J Arthrosc Rel Surg. 2022;38:2761–2766. 10.1016/j.arthro.2022.04.014 35550419

[60] Ranti D , Warburton AJ , Hanss K , Katz D , Poeran J , Moucha C . K‐means clustering to elucidate vulnerable subpopulations among medicare patients undergoing total joint arthroplasty. J Arthroplasty. 2020;35:3488–3497. 32739081 10.1016/j.arth.2020.06.063

[61] Rieke N , Hancox J , Li W , Milletarì F , Roth HR , Albarqouni S , et al. The future of digital health with federated learning. npj Dig Med. 2020;3:119. 10.1038/s41746-020-00323-1 PMC7490367 33015372

[62] Rouzrokh P , Khosravi B , Mickley JP , Erickson BJ , Taunton MJ , Wyles CC . THA‐net: a deep learning solution for next‐generation templating and patient‐specific surgical execution. J Arthroplasty. 2024;39(3):727–733. 37619804 10.1016/j.arth.2023.08.063

[63] Sagheb E , Ramazanian T , Tafti AP , Fu S , Kremers WK , Berry DJ , et al. Use of natural language processing algorithms to identify common data elements in operative notes for knee arthroplasty. J Arthroplasty. 2021;36:922–926. 33051119 10.1016/j.arth.2020.09.029 PMC7897213

[64] Shim E , Kim JY , Yoon JP , Ki S‐Y , Lho T , Kim Y , et al. Automated rotator cuff tear classification using 3D convolutional neural network. Sci Rep. 2020;10:15632. 32973192 10.1038/s41598-020-72357-0 PMC7518447

[65] Stojadinovic A , Kyle Potter B , Eberhardt J , Shawen SB , Andersen RC , Forsberg JA , et al. Development of a prognostic naïve bayesian classifier for successful treatment of nonunions. J Bone Jt Surg Am Vol. 2011;93:187–194. 10.2106/JBJS.I.01649 21248216

[66] Suzuki T , Maki S , Yamazaki T , Wakita H , Toguchi Y , Horii M , et al. Detecting distal radial fractures from wrist radiographs using a deep convolutional neural network with an accuracy comparable to hand orthopedic surgeons. J Digit Imaging. 2022;35:39–46. 34913132 10.1007/s10278-021-00519-1 PMC8854542

[67] Tavabi N , Pruneski J , Golchin S , Singh M , Sanborn R , Heyworth B , et al. Building large‐scale registries from unstructured clinical notes using a low‐resource natural language processing pipeline. Artif Intell Med. 2024;151:102847. 38658131 10.1016/j.artmed.2024.102847

[68] Tavabi N , Singh M , Pruneski J , Kiapour AM . Systematic evaluation of common natural language processing techniques to codify clinical notes. PLoS One. 2024;19:e0298892. 38451905 10.1371/journal.pone.0298892 PMC10919678

[69] Thirukumaran CP , Zaman A , Rubery PT , Calabria C , Li Y , Ricciardi BF , et al. Natural language processing for the identification of surgical site infections in orthopaedics. J Bone Jt Surg. 2019;101:2167–2174. 10.2106/JBJS.19.00661 PMC7002080 31596819

[70] Tibbo ME , Wyles CC , Fu S , Sohn S , Lewallen DG , Berry DJ , et al. Use of natural language processing tools to identify and classify periprosthetic femur fractures. J Arthroplasty. 2019;34:2216–2219. 31416741 10.1016/j.arth.2019.07.025 PMC6760992

[71] Tsai TL , Fridsma DB , Gatti G . Computer decision support as a source of interpretation error: the case of electrocardiograms. J Am Med Inform Assoc. 2003;10:478–483. 12807810 10.1197/jamia.M1279 PMC212785

[72] Van Eetvelde H , Mendonça LD , Ley C , Seil R , Tischer T . Machine learning methods in sport injury prediction and prevention: a systematic review. J Exp Orthop. 2021;8:27. 33855647 10.1186/s40634-021-00346-x PMC8046881

[73] Velasquez Garcia A , Bukowiec LG , Yang L , Nishikawa H , Fitzsimmons JS , Larson AN , et al. Artificial intelligence–based three‐dimensional templating for total joint arthroplasty planning: a scoping review. Int Orthop. 2024;48:997–1010. 38224400 10.1007/s00264-024-06088-6

[74] Wang H , Fu T , Du Y , Gao W , Huang K , Liu Z , et al. Scientific discovery in the age of artificial intelligence. Nature. 2023;620:47–60. 37532811 10.1038/s41586-023-06221-2

[75] Whiteside D , Martini DN , Lepley AS , Zernicke RF , Goulet GC . Predictors of ulnar collateral ligament reconstruction in major league baseball pitchers. Am J Sports Med. 2016;44:2202–2209. 27159303 10.1177/0363546516643812

[76] Wyles CC , Tibbo ME , Fu S , Wang Y , Sohn S , Kremers WK , et al. Use of natural language processing algorithms to identify common data elements in operative notes for total hip arthroplasty. J Bone Jt Surg. 2019;101:1931–1938. 10.2106/JBJS.19.00071 PMC7406139 31567670

[77] Yamada Y , Maki S , Kishida S , Nagai H , Arima J , Yamakawa N , et al. Automated classification of hip fractures using deep convolutional neural networks with orthopedic surgeon‐level accuracy: ensemble decision‐making with antero‐posterior and lateral radiographs. Acta Orthop. 2020;91:699–704. 32783544 10.1080/17453674.2020.1803664 PMC8023868

[78] Ye Z , Zhang T , Wu C , Qiao Y , Su W , Chen J , et al. Predicting the objective and subjective clinical outcomes of anterior cruciate ligament reconstruction: a machine learning analysis of 432 patients. Am J Sports Med. 2022;50:3786–3795. 36285651 10.1177/03635465221129870

[79] Yeo I , Klemt C , Melnic CM , Pattavina MH , De Oliveira BMC , Kwon Y‐M . Predicting surgical operative time in primary total knee arthroplasty utilizing machine learning models. Arch Orthop Trauma Surg. 2023;143:3299–3307. 35994094 10.1007/s00402-022-04588-x

[80] Yocum D , Reinbolt J , Weinhandl JT , Standifird TW , Fitzhugh E , Cates H , et al. Principal component analysis of knee joint differences between bilateral and unilateral total knee replacement patients during level walking. J Biomech Eng. 2021;143(11):111003. 34159353 10.1115/1.4051524

[81] Yu K‐H , Beam AL , Kohane IS . Artificial intelligence in healthcare. Nat Biomed Eng. 2018;2:719–731. 31015651 10.1038/s41551-018-0305-z

[82] Zhan H , Teng F , Liu Z , Yi Z , He J , Chen Y , et al. Artificial intelligence aids detection of rotator cuff pathology: a systematic review. Arthrosc J Arthrosc Rel Surg. 2024;40:567–578. 10.1016/j.arthro.2023.06.018 37355191

[83] Zhang K , Hu H , Philbrick K , Conte GM , Sobek JD , Rouzrokh P , et al. SOUP‐GAN: super‐resolution MRI using generative adversarial networks. Tomography. 2022;8:905–919. 35448707 10.3390/tomography8020073 PMC9027099

[84] Zhang Y , Weng Y , Lund J . Applications of explainable artificial intelligence in diagnosis and surgery. Diagnostics. 2022;12:237. 35204328 10.3390/diagnostics12020237 PMC8870992

[85] Zsidai B , Hilkert AS , Kaarre J , Narup E , Senorski EH , Grassi A , et al. A practical guide to the implementation of AI in orthopaedic research ‐ part 1: opportunities in clinical application and overcoming existing challenges. J Exp Orthop. 2023;10:117. 37968370 10.1186/s40634-023-00683-z PMC10651597

[86] Zsidai B , Kaarre J , Narup E , Hamrin Senorski E , Pareek A , Grassi A , et al. A practical guide to the implementation of artificial intelligence in orthopaedic research‐part 2: a technical introduction. J Exp Orthop. 2024;11:e12025. 38715910 10.1002/jeo2.12025 PMC11076014

